# miR-200b inhibits migration and invasion in non-small cell lung cancer cells via targeting FSCN1

**DOI:** 10.3892/mmr.2021.12218

**Published:** 2021-06-14

**Authors:** Peng Xiao, Wenliang Liu, Hui Zhou

Mol Med Rep 14: 1835-1840, 2016; DOI: 10.3892/mmr.2016.5421

Following the publication of this paper, it was drawn to the Editors’ attention by a concerned reader that certain of the Transwell assay data shown in Figs. 2C and 5C bore unexpected similarities to data appearing in different form in other articles by different authors. Owing to the fact that some of the contentious data in the above article had already been published elsewhere, or were already under consideration for publication, prior to its submission to *Molecular Medicine Reports*, the Editor has decided that this paper should be retracted from the Journal. After having been in contact with the authors, they agreed with the decision to retract the paper. The Editor apologizes to the readership for any inconvenience caused.

